# Lithium in patients with amyotrophic lateral sclerosis (LiCALS): a phase 3 multicentre, randomised, double-blind, placebo-controlled trial

**DOI:** 10.1016/S1474-4422(13)70037-1

**Published:** 2013-04

**Authors:** UKMND-LiCALS Study Group

## Abstract

**Background:**

Lithium has neuroprotective effects in cell and animal models of amyotrophic lateral sclerosis (ALS), and a small pilot study in patients with ALS showed a significant effect of lithium on survival. We aimed to assess whether lithium improves survival in patients with ALS.

**Methods:**

The lithium carbonate in amyotrophic lateral sclerosis (LiCALS) trial is a randomised, double-blind, placebo-controlled trial of oral lithium taken daily for 18 months in patients with ALS. Patients aged at least 18 years who had ALS according to the revised El Escorial criteria, had disease duration between 6 and 36 months, and were taking riluzole were recruited from ten centres in the UK. Patients were randomly assigned (1:1) to receive either lithium or matched placebo tablets. Randomisation was via an online system done at the level of the individual by block randomisation with randomly varying block sizes, stratified by study centre and site of disease onset (limb or bulbar). All patients and assessing study personnel were masked to treatment assignment. The primary endpoint was the rate of survival at 18 months and was analysed by intention to treat. This study is registered with Eudract, number 2008-006891-31.

**Findings:**

Between May 26, 2009, and Nov 10, 2011, 243 patients were screened, 214 of whom were randomly assigned to receive lithium (107 patients) or placebo (107 patients). Two patients discontinued treatment and one died before the target therapeutic lithium concentration could be achieved. 63 (59%) of 107 patients in the placebo group and 54 (50%) of 107 patients in the lithium group were alive at 18 months. The survival functions did not differ significantly between groups (Mantel-Cox log-rank χ^2^ on 1 df=1·64; p=0·20). After adjusting for study centre and site of onset using logistic regression, the relative odds of survival at 18 months (lithium *vs* placebo) was 0·71 (95% CI 0·40–1·24). 56 patients in the placebo group and 61 in the lithium group had at least one serious adverse event.

**Interpretation:**

We found no evidence of benefit of lithium on survival in patients with ALS, but nor were there safety concerns, which had been identified in previous studies with less conventional designs. This finding emphasises the importance of pursuing adequately powered trials with clear endpoints when testing new treatments.

**Funding:**

The Motor Neurone Disease Association of Great Britain and Northern Ireland.

## Introduction

Amyotrophic lateral sclerosis (ALS) is a neurodegenerative disease in which motor neurons in the brain and spinal cord degenerate, resulting in progressive paralysis ultimately leading to dependence on mechanical ventilatory support or death, usually within 3 years. Riluzole, a benzothiazole derivative, improves survival in patients with ALS;^1^ however, the effect is moderate and there remains a pressing need for more effective disease-modifying treatments.

Lithium has neuroprotective effects in cell^2^, ^3^, ^4^, ^5^, ^6^ and animal^7^ models of neurodegeneration, including ALS, although not in all studies.^8^, ^9^ In a positive study, transgenic ALS mice treated with lithium showed improved survival compared with wild-type mice treated with saline,^7^ and in a pilot study of lithium in patients with ALS there was a significant effect on survival in the lithium plus riluzole group compared with the riluzole only group.^7^ The design of that trial could be criticised because the method of randomisation was not stated, it was not placebo-controlled, and only 44 patients were included.^7^, ^10^ However, the reported difference in survival at 15 months (100% survival in the lithium plus riluzole group compared with 70% in the riluzole only group),^7^ taken together with the neuroprotective and neuroregenerative effects of lithium in the cell and animal models, suggested that a definitive randomised placebo-controlled trial was warranted. Therefore, a UK group of Motor Neurone Disease Association-supported ALS centres within the Dementias and Neurodegenerative Diseases Research Network (DeNDRoN) designed and undertook the lithium carbonate in amyotrophic lateral sclerosis (LiCALS) trial to test the hypothesis that lithium improves survival in ALS. Although several other trials were either in progress at the time or were planned, we knew that none had the same survival-based design, and we argued that a definitive answer was needed to show whether lithium has a biologically and clinically significant effect on survival and function and is safe and well tolerated in ALS.

## Methods

### Patients

LiCALS was a multicentre, double-blind, randomised, placebo-controlled trial undertaken at specialist ALS clinics at ten participating centres in the UK. The full protocol is described elsewhere.^11^ Eligible participants were adults aged at least 18 years who met the following criteria: possible, laboratory supported probable, probable, or definite ALS according to the revised version of the El Escorial criteria (the Airlie House Statement);^12^ an electromyogram compatible with ALS; disease duration of at least 6 months and no more than 36 months (inclusive); and receipt of riluzole treatment for at least 1 month before enrolment. Women of childbearing potential were excluded if pregnant, if breastfeeding, or if a urine pregnancy test before randomisation was not negative. Exclusion criteria included participation in another therapeutic study in the preceding 12 weeks, use of other investigational drugs, tracheostomy or other assisted ventilation in the preceding 3 months, an existing gastrostomy, a medical disorder that might interfere with diagnosis or functional assessment, hepatic or renal insufficiency, a major psychiatric disorder, clinically evident dementia, or allergy to lithium.

All participants gave written, informed consent to participate before screening. The study was ethically approved by the South East Research Ethics Committee, reference 09/H1102/15.

### Randomisation and masking

Randomisation was done via an online system based at the King's Clinical Trials Unit (CTU) at the Institute of Psychiatry (London, UK). Patients were randomised (1:1) to lithium or placebo at the level of the individual by block randomisation with randomly varying block sizes, stratified by study centre and site of disease onset (limb or bulbar). Active and placebo lithium tablets were identical in appearance, dimensions, mass, and disintegration time, and patients were prescribed up to three tablets daily, adjusted according to serum lithium concentrations. Active tablets contained 295 mg of lithium. The aim was to achieve therapeutic lithium concentrations, defined as 0·4–0·8 mmol/L. To maintain double-blind status while retaining the ability to monitor lithium concentrations, one physician from each site was unmasked but had no patient contact. The unmasked physician instructed the masked physicians and nurses to adjust the dose. Unmasked research nurses assisted unmasked physicians in the collection of laboratory results and in ensuring sign off by the physician administering lithium. Blood lithium concentrations were entered on a web-based electronic case report form system, which was accessible only to the study data manager and the unmasked physicians so that central monitoring of lithium concentrations was possible. The unmasked physician adjusted the lithium dose of patients in response to lithium concentrations and the King's CTU checked that this process was consistent and timely. For patients on placebo, sham dose adjustments were recommended to the unmasked physician by the CTU to avoid unmasking. Placebo dose adjustments were done by pairing patients in the lithium and placebo groups as they were randomised. Each time a patient in the lithium group was randomly assigned a group, the unmasked study data manager was alerted by the randomisation system when the next patient in any centre was randomly assigned to placebo. The data manager then monitored the dose adjustments of the patient in the lithium group who was randomised first and instructed the study physician of the paired patient on placebo to adjust the dose of study drug at the same time after randomisation as for the active patient and to the same dose adjustment. This process was monitored to ensure masked physicians and nurses remained masked throughout the trial (appendix). Adverse events were managed by clinicians masked to treatment allocation and serum lithium concentrations.

### Procedures

The primary outcome was death from any cause at 18 months, defined from date of randomisation and verified with documented evidence of death or of survival beyond 18 months in all cases. Secondary outcome measures, which comprised functional health status measured with the ALS functional rating scale-revised (ALSFRS-R), mental health state measured with the hospital anxiety and depression scale, and quality of life measured with the EuroQol (the EuroQoL group 5-dimension self-report questionnaire health state tariff and health evaluation scale), were assessed at baseline and 3, 6, 9, 12, 15, and 18 months. Reports of adverse events, whether related to the study drug or not, were collected and recorded at each timepoint. The masked physician at each site was responsible for deciding whether an adverse event was serious according to the Good Clinical Practice guidelines. Completed serious adverse event forms were then sent to the coordinating centre for review by the Chief Investigator. Non-serious adverse events were recorded in the electronic case report form alone.

### Statistical analysis

The sample size was based on detection of a difference in survival rates at 18 months using Fleiss's method for a proportion incorporating a continuity correction. Two groups of 110 patients would give 80% power to detect a difference of 17·5% in survival rates (65% *vs* 82·5%) assuming a two-sided type 1 error rate of 5%. The figure of 82·5% represents a treatment effect midway between a typical survival rate at 18 months of 65% and the 100% survival reported in the original positive lithium study.^7^

**Figure:**
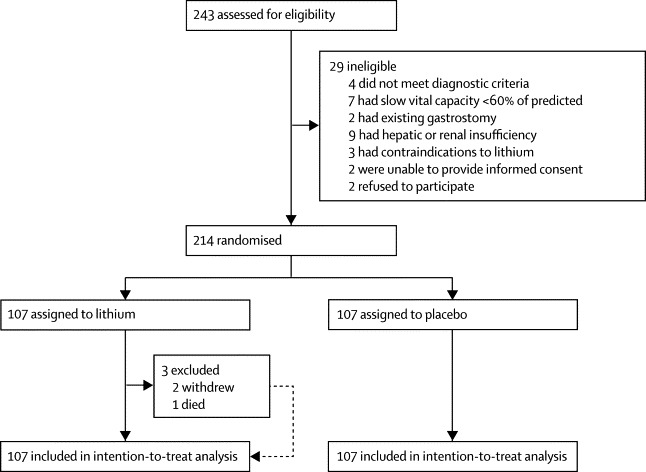
Trial profile

The primary analysis was of survival rates at 18 months in patients randomised to lithium treatment versus patients randomised to placebo by intention to treat, compared by the Mantel-Cox log-rank χ^2^ statistic. Originally, a test of survival proportions was planned, but clinical feedback suggested that survival over the entire study was important, and a log-rank test would be more in keeping with other studies of other drugs for treatment of ALS. This decision was made in August, 2010, before the masking was broken. This endpoint was a protocol amendment that was approved by the trial steering committee before the protocol was published (September 2011). Two further pre-specified analyses of the primary outcome measure were done: an intention-to-treat analysis of survival rates at 18 months using logistic regression with adjustment for randomisation strata, and a per-protocol analysis of time to death (censored at 18 months) using a Cox proportional hazards model. Patients were included in these analyses only if they had taken at least 75% of tablets prescribed in every quarter during participation.

Compliance was assessed using the number of tablets prescribed as recorded in the medication log compared with the number of tablets returned to the pharmacy at each study visit. The site nurses recorded the returns in the electronic case report form, site pharmacies recorded the returns independently in the pharmacy file, and the trial manager checked discrepancies between electronic case report form and pharmacy files during site visits, to ensure the correct returns were recorded on both, before drug returns were shipped back to the manufacturer for eventual destruction.

Rate of change in each of the secondary outcome measures was compared between groups using mixed models with variation between patients and variation between occasions nested within patients treated as random effects. Time in years was treated as a continuous variable. Results, adjusted for differences between randomisation strata, are given in the form of differences in rate of change with corresponding 95% CIs between patients assigned to lithium and those assigned to placebo. This approach was extended to assess the effect of missing data^13^ by joint modelling of the survival data (with a Cox proportional hazards model) and the secondary outcomes (with mixed models similar to those described earlier). The Cox model was built by forward stepwise regression, but backward elimination gave the same model. The variables included were El Escorial category, age of onset, slow vital capacity, and sex. An effect of lithium was then added to the model. Variables excluded were handedness, site of symptom onset, time between onset and diagnosis, and pulse rate. Within both of these models, an additional latent variable was included that can be conceptualised as the propensity to experience poor outcomes. The inclusion of this latent variable is common to both models and allows us to adjust our estimates of the treatment effect to allow for the different rates of dropout in each group. Both models were estimated simultaneously, thus maximising the joint likelihood over both the survival and repeated measures data. Results are given in the form of the difference in rate of change of each of the secondary outcomes with corresponding 95% CIs.

Time to first serious adverse event was analysed with a Cox proportional hazards model. We calculated hazard ratios (risk of a serious adverse event) and associated 95% CIs for patients assigned to lithium compared with patients assigned to placebo for all serious adverse events and for all serious adverse events excluding death. For quality of life scores, data imputation was undertaken provided at least half of the items in any scale had been completed; the imputed missing value was defined as the mean value of the non-missing items.

This study is registered with Eudract, number 2008-006891-31.

### Role of the funding source

The funding source had no role in study design, data collection, data analysis, data interpretation, writing of the report, or the decision to submit for publication. The corresponding author had full access to all data in the study, and the manuscript was written by the corresponding author with assistance from other members of the writing committee. The corresponding author had final responsibility for the decision to submit for publication.

## Results

The figure shows the trial profile. Between May 26, 2009, and Nov 10, 2011, 243 patients were screened, 214 of whom were recruited from ten centres (appendix) and were randomly assigned to lithium (n=107) or to placebo (n=107). The groups were well balanced for demographic and clinical characteristics at baseline (table 1). Compliance was good, with 140 patients (65%) taking at least 75% of the prescribed drug in every quarter. Compliance was better in the placebo group (71%) than in the lithium group (60%). During the course of the study, 65 patients withdrew from the study drug (23 on placebo and 42 on lithium), 41 owing to adverse events (12 on placebo and 29 on lithium), seven because they were no longer able to travel to the centre (three on placebo and four on lithium), and 17 for other reasons (eight on placebo and nine on lithium).

**Table 1:** Demographic and baseline characteristics

		**Placebo (n=107)**	**Lithium (n=107)**
Women		30 (28%)	36 (34%)
Ethnic origin, white		104 (97%)	106 (99%)
Age at recruitment (years)		59·5 (11·5)	59·7 (9·9)
Time from onset of symptoms to diagnosis (weeks)		47·0 (27·8)	46·3 (26·3)
Time from diagnosis to recruitment (weeks)		36·8 (29·0)	34·0 (28·1)
Disease duration at start of study (weeks)		80·3 (33·8)	83·8 (36·0)
Bulbar site of onset		24 (22%)	23 (22%)
Sporadic type of onset		83 (78%)	84 (79%)
Right handedness		94 (88%)	92 (86%)
El Escorial diagnostic category			
	Clinically definite ALS	41 (38%)	41 (38%)
	Clinically probable ALS	43 (40%)	37 (35%)
	Clinically probable laboratory supported ALS	18 (17%)	20 (19%)
	Clinically possible ALS	5 (5%)	9 (8%)
Vital capacity in spirometry (% predicted)		89·3 (17·0)	93·3 (18·5)
Pulse rate (beats per min)		74·4 (12·2)	76·7 (13·9)
Systolic blood pressure (mm Hg)		135·0 (16·9)	132·6 (15·8)
Diastolic blood pressure (mm Hg)		84·1 (12·0)	83·6 (15·3)
Time on riluzole at entry to study (days)		198·5 (177·7)	209·6 (192·0)
ALS functional rating scale-revised		38·64 (5·72)	38·20 (5·66)
HADS anxiety		4·46 (3·76)	4·59 (3·37)
HADS depression		4·00 (3·10)	3·89 (2·84)
EuroQoL health state tariff		0·59 (0·28)	0·59 (0·30)
EuroQol health evaluation		70·07 (19·48)	68·50 (18·50)

Data are number (%) or mean (SD). ALS=amyotrophic lateral sclerosis. HADS=hospital anxiety and depression scale.

Of 107 patients randomly assigned to treatment with lithium carbonate, 104 (97%) had at least one blood lithium concentration measurement in the therapeutic range (0·4–0·8 mmol/L), with the mean number of such measurements being 6·6 (SD 2·9; appendix). Of the three patients who did not achieve the therapeutic range, one withdrew after becoming pregnant, one withdrew after an adverse event 1 week into the study, and one had a serious adverse event leading to death before the therapeutic range could be achieved.

Other factors that potentially influenced survival did not differ significantly between groups. By the end of the 18 month follow-up period or at time of death, 36 (34%) patients in the placebo group and 25 (23%) in the lithium group had received percutaneous endoscopic gastrostomy (relative risk 0·69, 95% CI 0·45–1·07). 29 (27%) patients in the placebo group and 27 (25%) in the lithium group had received non-invasive ventilation (relative risk 0·93, 95% CI 0·59–1·46).

63 (59%) patients in the placebo group (56 on study drug and seven off study drug) and 54 (50%) patients in the lithium group (41 on study drug and 13 off study drug) were alive at 18 months. The survival functions did not differ significantly between groups (Mantel-Cox log-rank χ^2^ statistic on 1 df=1·64; p=0·20). In a post-hoc analysis, after adjusting for study centre and site of onset using a Cox proportional hazards model, the estimated hazard ratio (lithium *vs* placebo) was 1·35 (95% CI 0·90–2·02). The corresponding result in patients who complied with their treatment (64 in the lithium group and 76 in the placebo group) was 1·40 (95% CI 0·83–2·34). The relative odds of survival at 18 months (lithium *vs* placebo) adjusted for centre and site of onset was 0·71 (95% CI 0·40–1·24). Therefore, there was no evidence that treatment with lithium influenced survival in this patient population.

Table 2 lists the mean scores for the secondary outcome measures. The analytical strategy was to compare the rate of change of health status in the two groups by fitting statistical models that allowed for repeated measures within individuals, for differences between randomisation strata, and for loss to follow-up (appendix). There was a marked deterioration in functional health status in both treatment groups.

**Table 2:** Secondary outcome measures, by time since randomisation

	**0 months**	**3 months**	**6 months**	**9 months**	**12 months**	**15 months**	**18 months**
**ALS functional rating scale-revised**							
Placebo	38·64 (5·72)	35·27 (8·09)	33·43 (8·74)	32·13 (8·42)	30·20 (8·90)	28·88 (9·14)	28·54 (9·27)
Lithium	38·20 (5·66)	36·17 (6·65)	32·40 (8·24)	30·04 (8·55)	28·31 (9·50)	29·47 (10·23)	29·32 (9·96)
HADS anxiety*							
Placebo	4·46 (3·76)	4·33 (3·62)	4·19 (3·76)	4·53 (3·86)	4·67 (4·05)	4·54 (4·03)	3·50 (3·54)
Lithium	4·59 (3·37)	5·03 (4·19)	5·30 (4·08)	5·09 (3·90)	5·96 (4·42)	5·26 (4·05)	4·55 (4·26)
HADS depression*							
Placebo	4·00 (3·10)	4·36 (3·06)	4·53 (3·65)	4·74 (3·30)	5·05 (3·62)	5·67 (3·96)	4·71 (3·76)
Lithium	3·89 (2·84)	4·83 (3·32)	5·61 (3·66)	5·80 (3·45)	5·88 (3·60)	5·03 (3·60)	5·17 (3·92)
**EuroQoL health state tariff**							
Placebo	0·59 (0·28)	0·50 (0·31)	0·46 (0·34)	0·42 (0·33)	0·37 (0·34)	0·33 (0·32)	0·33 (0·35)
Lithium	0·59 (0·30)	0·54 (0·35)	0·42 (0·38)	0·35 (0·35)	0·30 (0·38)	0·32 (0·39)	0·30 (0·40)
**EuroQoL health evaluation**							
Placebo	70·07 (19·48)	63·74 (22·32)	64·89 (20·21)	64·60 (21·31)	61·97 (21·36)	59·43 (21·74)	61·95 (23·97)
Lithium	68·50 (18·50)	64·09 (22·22)	61·17 (21·37)	60·14 (21·11)	57·03 (24·48)	56·40 (24·07)	56·36 (22·86)

Data are mean (SD). ALS=amyotrophic lateral sclerosis. HADS=hospital anxiety and depression scale.

* For the two HADS scales, a higher score corresponds to poorer outcome; for all other outcomes a higher score corresponds to better outcome.

In the unadjusted analysis for the ALSFRS-R—a functional scale from 0 to 48, where 48 is maximal function—the annual rate of change was −9·31 (95% CI −10·5 to −8·58) in the placebo group and −9·50 (−10·31 to −8·70) in the lithium group (appendix). The difference between these rates was −0·19 (−1·28 to 0·90) and was not significant. Based on joint modelling, the rate of change in ALSFRS-R adjusted for survival was −9·47 (95% CI −10·98 to −8·46) in the placebo group and −9·75 (−11·62 to −8·47) in the lithium group. These increases in the estimated magnitude of change are consistent with the assumption that those patients who were lost to follow-up were those with the poorest functional status. As in the unadjusted analysis, the difference between these rates of decline in this adjusted analysis was not statistically significant (difference −0·28, 95% CI −2·40 to 1·67), and this remained non-significant after adjustment for strata (appendix).

Anxiety scores increased over the period of the study but the increases were small and the difference between groups was not significant (appendix). Patients in both groups became more depressed over time, but the difference in the rate of change was not significant (estimated difference based on the joint model adjusting for randomisation strata was 0·29, 95% CI 0·33 to 1·02). Similarly, quality of life deteriorated over time in both groups but the difference in the rate of change was not significant.

117 patients had at least one serious adverse event during the 18 month follow-up period (56 patients in the placebo group and 61 patients in the lithium group; table 3). Four patients had three recorded serious adverse events, 30 patients had two, and 83 patients had one. The estimated hazard ratios (lithium *vs* placebo) were 1·14 (95% CI 0·79–1·65) for all serious adverse events and 0·84 (0·52–1·36) for events excluding death, neither of which were significant.

**Table 3:** Serious adverse events

	**Placebo**	**Lithium**	**Total**
None	51	46	97
1 event	41	42	83
2 events	14	16	30
3 events	1	3	4
Total	107	107	214

Data are number of patients. One patient had a serious adverse event between two screening visits and before signing the consent form. Because this event occurred before randomisation it has been excluded from the analysis. The relative risk of a serious adverse event (including death) in the lithium group compared with the placebo group was 1·09 (95% CI 0·58–1·39).

## Discussion

In this double-blind, randomised controlled trial of lithium versus placebo in ALS using a survival design, we found that there was no evidence that treatment with lithium resulted in an increase in survival at 18 months (panel). There was a marked deterioration in functional health status and quality of life, with an associated increase in depression and anxiety over time in patients assigned to either treatment group.

Panel **Research in context**

**Systematic review**

We searched PubMed for reports published before Dec 1, 2012, using the following terms: “lithium” and “amyotrophic lateral sclerosis”, “motor neuron disease”, “motor neurone disease”, “ALS”, or “MND”. We included randomised, placebo-controlled trials and trials of other designs in amyotrophic lateral sclerosis (ALS) or a related disorder that involved lithium. We identified six previous trials, three of which were randomised, placebo-controlled trials,^14^, ^15^, ^16^ one that was randomised but not placebo controlled,^7^ one that used historical controls,^17^ and one that used self-reporting by patients.^18^ We noted that all six trials used different, non-traditional methods for design or analysis or both. We did a randomised, double-blind, placebo-controlled trial with a primary endpoint of survival rates at 18 months in 214 patients with ALS, half assigned to lithium treatment and half assigned to placebo.

**Interpretation**

We noted no difference in survival between placebo and lithium groups. This study confirms the absence of benefit of lithium for treatment of ALS.

The first reported study of lithium in patients with ALS^7^ examined 44 patients, 16 given lithium and riluzole and 28 given riluzole alone. There were no deaths at 15 months in the riluzole and lithium group compared with eight (29%) in the riluzole only group. Furthermore, patients in the lithium group showed little progression on functional measures compared with typical progression in the control group. This study came after a study of mutant *SOD1* transgenic mice that showed compelling evidence for a neuroprotective effect of lithium in ALS.^7^ The study was not placebo controlled and was small, although adequately powered to detect an effect similar to that noted in the mice.^7^ Our study was powered to detect an effect on survival but, additionally, neither standard analysis of function nor joint analysis of function and survival, which accounts for the loss of those dying with worse function, showed any benefit of lithium.

Similar findings have been noted in other studies (table 4), although none of these had the traditional design of the present study, in which survival was used as an endpoint in a fixed-duration study with two treatment groups. Thus, despite several studies that failed to show evidence of benefit for lithium therapy in ALS, none was a traditionally designed double-blind, placebo-controlled, randomised trial with a primary endpoint of survival, and arguably none of these trials could robustly resolve whether lithium could have a small but biologically significant effect on ALS progression, as measured by the gold standard endpoint for phase 3 studies, survival. Although our study is underpowered for detection of a small change in survival (eg, 5–10%, as detected for riluzole^1^), we accepted this limitation as a pragmatic compromise for an academic-led trial with limited financial resources and without the need for regulatory approval had we detected a beneficial effect. Adjusting for study centre and site of onset, the relative odds of survival at 18 months (lithium *vs* placebo) was 0·71 (95% CI 0·40–1·24). Our projected survival rates of 65% and 82·5% in an achieved sample of 214 patients would correspond to an odds ratio of 2·54—significantly higher than the upper limit of 1·24 in our estimate. We can therefore be confident that if there is any effect of treatment with lithium on survival, it is very much lower than that hypothesised.

**Table 4:** Previous studies of lithium treatment in patients with amyotrophic lateral sclerosis

	**Study design**	**Study size**	**Outcome**	**Comments**
Aggarwal et al (2010)^14^	Sequential, time-to-event, futility	88	Stopped early (mean duration 5·4 months) because futility boundary (p=0·68) was crossed	Design would not detect delayed benefit (eg, if lithium takes 1 year to have an effect)
Chio et al (2010)^15^	Single blind	171	Stopped early by data monitoring and ethics committee because 117 patients discontinued	Provides weak evidence in any direction
Miller et al (2011)^17^	Historical controls, unmasked	107	No benefit of lithium therapy	Control individuals were not randomly selected from the same population as those treated and cannot be regarded as truly matched
Wicks et al (2011)^18^	Observational using self-reported patient data	447	No benefit of lithium therapy	No placebo, control individuals selected by patient-matching algorithm rather than randomised
Verstraete et al (2012)^16^	Randomised sequential	133	No benefit of lithium therapy	Design potentially prevents detection of late effect of treatment

Studies included in the table are those that aimed to replicate the findings of the first study on lithium treatment in patients with amyotrophic lateral sclerosis.^7^

Studies of function in clinical trials of ALS are difficult because patients with the worst function are most likely to die and no longer contribute scores to the mean; any decline is masked and there is reduced power to detect an effect. We have approached this problem in two ways. First, we analysed the outcomes in patients who did not survive until the study end, confirming outcomes were indeed poorer. Second, we adjusted for loss to follow-up, confirming that this adjustment increases the estimated annual change in functional scores, mental health scores, and quality of life. By undertaking a joint analysis of survival and function, we were able to control for the loss to follow-up in the comparison between treatment groups and showed no benefit of treatment. Further studies are needed to identify the optimum method for analysis of functional scores in trials of treatments in ALS.

In this phase 3, randomised, placebo-controlled, double-blind trial of lithium therapy in ALS, we did not find evidence of benefit, but nor were there safety concerns, which had been identified in previous studies with less conventional designs.^14^ This finding emphasises the importance of pursuing adequately powered trials with clear endpoints when testing new treatments, bearing in mind that a trial tests biologically important hypotheses as well as clinical efficacy. Previous lithium trials could not adequately address this issue, but our results suggest that we can now be confident that lithium at these serum concentrations does not significantly influence disease progression, as assessed either by a validated functional measure (ALFRS-R) or by survival.

Correspondence to: Prof Ammar Al-Chalabi, Department of Clinical Neuroscience, Institute of Psychiatry, King's College London, London SE5 8AF, UK **ammar.al-chalabi@kcl.ac.uk**
