# Supplementary material for: Lithium in patients with amyotrophic lateral sclerosis (LiCALS): a phase 3 multicentre, randomised, double-blind, placebo-controlled trial
Source: Lancet Neurol. 2013 Apr;12(4):339–45. doi: 10.1016/S1474-4422(13)70037-1 (PMC3610091; doi:10.1016/S1474-4422(13)70037-1)
Supplement: Supplementary appendix [file mmc1.pdf]

THE LANCET Neurology

Supplementary webappendix

This webappendix formed part of the original submission and has been peer reviewed. We post it as supplied by the authors.

Supplement to: UKMND-LiCALS Study Group. Lithium in patients with amyotrophic lateral sclerosis (LiCALS): a phase 3 multicentre, randomised, double-blind, placebo-controlled trial. *Lancet Neurol* 2013; published online Feb 27. http://dx.doi.org/10.1016/S1474-4422(13)70037-1.

Web extra material

		Treatment allocation		Total
		Control	$LiCO_3$	
**Clinician guess**	**I strongly think he/she was prescribed active medication**	1	6	7
	**I think he/she was prescribed active medication**	35	36	71
	**I think he/she was prescribed placebo**	35	22	57
	**I strongly think he/she was prescribed placebo**	5	0	5
**Total**		76	64	140

Table S1. Clinician guess of treatment allocation

		Treatment allocation		Total
		Control	$LiCO_3$	
**Patient guess**	**I strongly think I was prescribed active medication**	4	12	16
	**I think I was prescribed active medication**	18	17	35
	**I think I was prescribed placebo**	27	13	40
	**I strongly think I was prescribed placebo**	17	13	30
**Total**		66	55	121

Table S2. Patient guess of treatment allocation

	Treatment allocation		
**Centre**	**Placebo**	**Lithium**	**Total**
**Birmingham**	14	12	26
**KCL**	10	9	19
**Liverpool**	12	13	25
**Manchester**	11	11	22
**NHNN**	11	11	22
**Newcastle**	9	9	18
**Oxford**	13	12	25
**Plymouth**	9	10	19
**Preston**	7	9	16
**Sheffield**	11	11	22
	107	107	214

Table S3. Recruitment from each centre

n	Frequency	Percent
0	3	2.8
1	3	2.8
2	5	4.7
3	8	7.5
4	4	3.7
5	11	10.3
6	12	11.2
7	16	15.0
8	14	13.1
9	12	11.2
10	13	12.1
11	4	3.7
12	1	.9
13	1	.9
Total	107	100.0

Table S4. The number of measurements within the therapeutic range.
For the 104 patients achieving therapeutic range, the proportion of subsequent measurements achieving therapeutic range varied from 15% to 100% with a mean of 82.1%. Thus the majority of patients spent the majority of time in the therapeutic range. 25 patients (23.4%) had at least one measurement above the recommended upper threshold of 0.8mmol/L. Typically this was a single value that occurred while the appropriate dose for a patient was being ascertained.

Measure	Annual rate of change in health status – Separate longitudinal analysis											
	Unadjusted						Adjusted for randomisation strata					
	Control		$LiCO_3$		Difference		Control		$LiCO_3$		Difference	
		95%CI		95%CI		95%CI		95%CI		95%CI		95% CI
**ALSFRS-R**	-9·31	-10·05, -8·58	-9·50	-10·31, -8·70	-0·19	-1·28, 0·90	-9·31	-10·04, -8·57	-9·52	-10·32, -8·71	-0·21	-1·30, 0·88
**HADS anxiety**	0·12	-0·28, 0·53	0·52	0·07, 0·96	0·40	-0·21, 1·00	0·13	-0·28, 0·53	0·52	0·08, 0·97	0·40	-0·21, 1·00
**HADS depression**	1·27	0·92, 1·62	1·53	1·15, 1·91	0·26	-0·26, 0·78	1·27	0·92, 1·62	1·54	1·16, 1·93	0·27	-0·25, 0·79
**EQ5-D**	-0·26	-0·29, -0·22	-0·29	-0·32, -0·25	-0·03	-0·08, 0·02	-0·26	-0·29, -0·22	-0·29	-0·33, -0·25	-0·03	-0·08, 0·02
**EuroQol**	-8·88	-11·28, -6·48	-11·83	-14·44, -9·21	-2·94	-6·49, 0·60	-8·88	-11·27, -6·48	-11·97	-14·58, -9·36	-3·10	-6·64, 0·45

Table S5. Separate longitudinal analysis of rate of change in functional health status, quality of life and mental health state

Measure	Annual rate of change in health status – After joint modelling with survival data											
	Adjusted only for survival						Adjusted for survival and randomisation strata					
	Control		$LiCO_3$		Difference		Control		$LiCO_3$		Difference	
		95%CI		95%CI		95%CI		95%CI		95%CI		95% CI
**ALSFRS-R**	-9·47	-10·98, -8·46	-9·75	-11·62, -8·47	-0·28	-2·40, 1·67	-9·44	-10·93, -8·44	-9·74	-11·57, -8·44	-0·30	-2·43, 1·69
**HADS anxiety**	0·15	-0·29, 0·68	0·55	-0·11, 1·15	0·41	-0·40, 1·02	0·14	-0·30, 0·68	0·54	-0·12, 1·15	0·40	-0·40, 1·02
**HADS depression**	1·31	0·87, 1·79	1·58	1·08, 2·04	0·27	-0·36, 1·01	1·31	0·87, 1·81	1·60	1·09, 2·07	0·29	-0·33, 1·02
**EQ5-D**	-0·26	-0·33, -0·22	-0·30	-0·36, -0·24	-0·03	-0·12, 0·04	-0·26	-0·33, -0·22	-0·30	-0·36, -0·24	-0·04	-0·12, 0·04
**EuroQol**	-9·26	-13·54, -6·80	-12·35	-17·93, -7·86	-3·09	-9·78, 1·51	-9·21	-13·46, -6·73	-12·49	-18·20, -8·00	-3·29	-9·86, 1·30

Table S6. Joint modelling of functional health status, quality of life and mental health state with survival data
